# Identification of Two Novel LAMA2 Mutations in a Chinese Patient with Congenital Muscular Dystrophy

**DOI:** 10.3389/fgene.2018.00043

**Published:** 2018-02-13

**Authors:** Jing Zhou, Jianxin Tan, Dingyuan Ma, Jingjing Zhang, Jian Cheng, Chunyu Luo, Gang Liu, Yuguo Wang, Zhengfeng Xu

**Affiliations:** Department of Prenatal Diagnosis, State Key Laboratory of Reproductive Medicine, The Affiliated Obstetrics and Gynecology Hospital of Nanjing Medical University, Nanjing Maternity and Child Health Care Hospital, Nanjing, China

**Keywords:** merosin-deficient CMD type 1A, laminin subunit alpha 2, mutations, aberrant splicing

## Abstract

Merosin-deficient CMD type 1A (MDC1A), caused by mutations of laminin subunit alpha 2 (LAMA2), is a predominant subtype of congenital muscular dystrophy (CMD). Herein, we described a Chinese patient with MDC1A who was admitted to hospital 17 days after birth because of marasmus and feeding difficulties. Mutations were identified by targeted capture and next generation sequencing (NGS) and further confirmed by Sanger sequencing. Paternity was confirmed by short tandem repeat analysis. Physical examination showed malnutrition, poor suck and appendicular hypotonia. Her serum CK levels were 2483 and 1962 U/L at 2 and 4 months of age, respectively. Brain magnetic resonance imaging performed at 1 month of age presented hyperintensity on T2-weighted images, T1-weighted images in parietal and occipital lobes, and diffusion-weighted image (DWI) as well as hypointensity on fluid attenuated inversion recovery (FLAIR) image; however, the cerebellum and corpus arenaceum were normal. At 7 months of age, delayed developmental milestones were observed, and she failed to turn her body over and raise her head up. A point mutation (c.1782+2T > G) and a frameshift duplication (c.8217dupT) in the LAMA2 gene were identified by targeted capture and NGS and further confirmed by Sanger sequencing. Moreover, genotyping with multiple short tandem repeat markers confirmed paternity to demonstrate that the point mutation is *de novo*. The frameshift duplication (c.8217dupT), inherited from her mother, was predicted to cause a substitution of Pro (P) to Ser (S) at the 2740th amino-acid residue and generate a prematurely truncated protein. The *in silico* analysis suggests that the mutation (c.1782+2T > G) may lead to aberrant splicing of LAMA2. Our case further confirms the heterogeneous clinical spectrum of MDC1A and presents two novel LAMA2 mutations to expand the mutation spectrum of MDC1A.

## Introduction

The congenital muscular dystrophies (CMDs) are a highly heterogeneous group of neuromuscular disorders with preferentially autosomal recessive inheritance (Philpot et al., 1995). Merosin-deficient CMD type 1A (MDC1A), caused by mutations in the LAMA2 gene, constitutes approximately 50% of total CMD cases and is characterized by hypotonia within the first several months of life, increased levels of serum creatine kinase (CK), multiple joint contractures, and white matter abnormalities (Herrmann et al., 1996; Cohn et al., 1998; Pegoraro et al., 1998). The LAMA2 gene spans 65 exons and encodes the laminin-α2 chain, which assembles with laminin-β1 and -γ1 to form laminin-211 in skeletal muscles (Colognato and Yurchenco, 2000). The mutations within the LAMA2 gene, without any identified hotspots, lead to complete or partial deficiency of laminin-α2. Patients with a partial deficiency of laminin-α2 exhibit a milder phenotype, while a complete deficiency of the protein always results in severe phenotypes, such as white matter alterations (Cohn et al., 1998). This is explained by the nature of individual mutations, which disrupts the assembly of laminin-α2 with other basal lamina chains (Gawlik and Durbeej, 2011). MDC1A is predominantly reported in European countries, but only a number of cases have been described in Asia (Miyagoe-Suzuki et al., 2000; Xiong et al., 2015). Herein, we report a Chinese patient with MDC1A carrying two novel mutations in the LAMA2 gene. Furthermore, our *in silico* analysis suggests that one of the mutations is likely to cause aberrant splicing of LAMA2.

## Case Presentation

**FIGURE 1:**
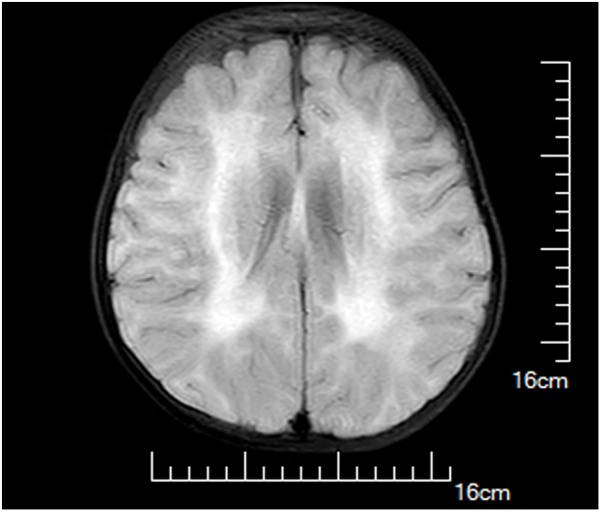
Brain magnetic resonance images of the proband at 1 month of age. Hyperintensity on T2-weighted images, T1-weighted images in parietal and occipital lobes, and diffusion-weighted image (DWI) as well as hypointensity on fluid attenuated inversion recovery (FLAIR) image were observed.

A female infant, the first child of healthy non-consanguineous Chinese parents, was born at 40+6 weeks of gestation by spontaneous vaginal delivery after an uneventful pregnancy. At birth, her weight was 3270 g, and Apgar scores were 10 at 1 and 5 min. She was admitted to our hospital 17 days after birth because of marasmus and feeding difficulties. On admission, physical examination showed malnutrition, poor suck, and appendicular hypotonia. Her serum CK levels were dramatically increased (2483 and 1962 U/L at 2 and 4 months of age, respectively). Brain magnetic resonance imaging performed at 1 month of age presented hyperintensity on T2-weighted images, T1-weighted images in parietal and occipital lobes, and DWI as well as hypointensity on FLAIR image; however, the cerebellum and corpus arenaceum were normal (**Figure 1**). At 7 months of age, delayed developmental milestones were observed, and she failed to turn her body over and raise her head up.

**FIGURE 2:**
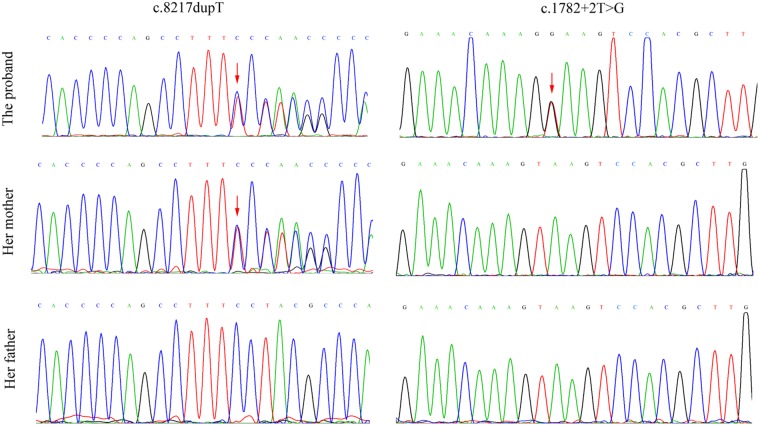
Mutation analysis and identification of LAMA2 gene in the proband and her parents. The frameshift duplication (c.8217dupT) was found in the proband and her mother, but the point mutation (c.1782+2T > G) was only observed in the proband. Red arrows indicate the heterozygous point mutations.

**FIGURE 3:**
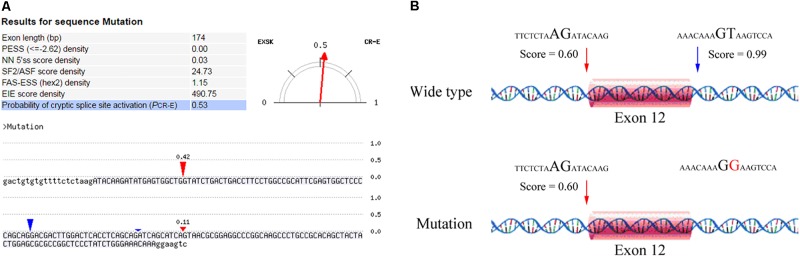
*In silico* analysis of the point mutation (c.1782+2T > G) in LAMA2 gene. **(A)** A screenshot of CRYP-SKIP analysis results. The input sequences include exon 12 (upper letters) and the corresponding flanking intron sequences (lower letters). The predictor variables analyzed by CRYP-SKIP are summarized in the table [putative exon splicing silencers (PESS); neural network 5′ splice sites (NN 5′ss); the most important SR protein (SF2/ASF)]. The predicted cryptic donor splice site and the predicted acceptor site are shown in red and blue vertical marks, respectively. **(B)** The results analyzed by BDGP prediction. An acceptor site and a donor site are indicated in red and blue arrows, respectively.

After counseling with a clinical geneticist, genetic analysis for neuromuscular disorders was carried out. The informed consent was obtained from her parents, and this study was approved by the Ethics Committee of Nanjing Maternity and Child Health Care Hospital. Written and signed informed consent for publishing the case report was also obtained from the patient’s guardians. Peripheral blood samples were collected from the proband and her parents, and genomic DNA was extracted from peripheral blood lymphocytes by using an Automated Nucleic Acid Extractor (RBCBioscience, New Taipei City, Taiwan). Targeted next generation sequencing (NGS) of 270 neuromuscular disease-associated genes was performed in the proband by using Ion Torrent Personal Genome Machine (PGM) platform (Thermo Fisher Scientific, South San Francisco, CA, United States). As a result, we identified a heterozygous point mutation (c.1782+2T > G) and a heterozygous frameshift duplication (c.8217dupT) in the LAMA2 gene (reference sequence: NM_000426.3), which were further confirmed by Sanger sequencing (**Figure 2**). Both variants are absent in Leiden Open Variation Database^1^. The duplication (c.8217dupT) was inherited from her mother, but the point mutation (c.1782+2T > G) was not observed in her parents. Paternity was confirmed by short tandem repeat analysis to demonstrate that the point mutation (c.1782+2T > G) is *de novo*. The duplication (c.8217dupT) was predicted to cause a frameshift mutation, which results in a substitution of Pro (P) to Ser (S) at the 2740th amino-acid residue and generates a prematurely truncated protein. The length of the encoded peptide chain was reduced from 3122 to 2778 (p. Pro2778SerfsX) amino-acid residues. Sequence analysis showed that the point mutation (c.1782+2T > G) is located at the exon–intron boundary, a potential splicing donor site. Therefore, *in silico* splicing analysis of this variation was performed by using two different web-based programs, CRYP-SKIP^2^ and Berkeley Drosophila Genome Project (BDGP)^3^. Mutations in splicing sites generally lead to exon skipping or activation of cryptic splice sites. The CRYP-SKIP analysis showed that the point mutation (c.1782+2T > G) may cause skipping of exon 12 or generation of a cryptic donor site, with probability of 0.47 and 0.53, respectively (**Figure 3A**). Meanwhile, BDGP predicted an acceptor site (score = 0.60) and a donor site (score = 0.99) at the flanking region of exon 12. However, the point mutation (c.1782+2T > G) resulted in the inactivation of the original splice donor site (**Figure 3B**). Our *in silico* splicing analysis suggests that the point mutation (c.1782+2T > G) is likely to cause aberrant splicing of LAMA2 mRNA. Taken together, our analysis implies that the two mutations in the LAMA2 gene may be the pathogenic mutations responsible for the phenotype in this patient.

## Discussion

MDC1A, an autosomal recessive disorder, is caused by mutations in the LAMA2 gene and represents the predominant subtype of CMD (Durbeej, 2015). Typical presentations of MDC1A include absence of the laminin α-2 chain around muscle fibers, increased levels of serum CK, and white matter abnormalities (Gawlik and Durbeej, 2011). However, it is still a challenge for clinicians to obtain a precise molecular diagnosis using conventional genetic methods because of the clinical and genetic heterogeneity of this disorder. Recently, targeted NGS technology is emerging as a powerful genetic tool and has been successfully used to identify causal genes in various genetic disorders (Simon et al., 2015). In the present study, we used targeted NGS of 270 neuromuscular disease-associated genes and identified two novel LAMA2 mutations in a Chinese patient with MDC1A. Our further *in silico* analysis suggests that the two variations are possible pathogenic mutations in this case.

MDC1A is previously diagnosed on the basis of clinical presentations, including severe congenital hypotonia, high serum CK levels, white matter alternations, as well as deficiency of merosin expression in biopsied muscle (Durbeej, 2015). Muscle biopsy appears to be an indispensable procedure to confirm the diagnosis of CMD. Nonetheless, recent evidence suggests that molecular genetic analysis may be an acceptable alternative to muscle biopsy if the clinical phenotypes support the diagnosis of CMD (He et al., 2013; Harris et al., 2017). In our case, the proband was suspected to have CMD owing to appendicular hypotonia, high serum CK levels, and white matter abnormalities. Therefore, we performed molecular genetic analysis and identified two novel LAMA2 mutations, which were predicted to be the pathogenic mutations. Finally, the proband was diagnosed with MDC1A in the absence of muscle biopsy. However, further studies are required to investigate the effects of these mutations on merosin protein.

To date, approximately 90 mutations of LAMA2 have been described, without any mutational hotspots (Beytia Mde et al., 2014). In the present case, we identified heterozygous frameshift duplication (c.8217dupT), which is located in exon 58 and was predicted to generate a prematurely truncated protein. The G domain (exons 46–64) at the C-terminus of merosin is involved in the linkage between the extracellular matrix and the dystrophin-glycoprotein (Mendell et al., 1998). This may explain why our case displays a severe phenotype. Since the autosomal recessive inheritance for MDC1A, the patient should carry two pathogenic variations in the LAMA2 gene. Meanwhile, we also identified a *de novo* point mutation (c.1782+2T > G) in this case, which was predicted to cause aberrant splicing of LAMA2 mRNA. Similarly, a number of mutations that cause abnormal splicing of LAMA2 have reported in MDC1A patients. For instance, Siala et al. (2007) reported a splicing mutation in the LAMA2 gene resulting in exon skipping and dramatic decrease of mRNA level in a severe MDC1A patient. However, our prediction needs to be validated by *in vitro* and *in vivo* studies.

Previous reports have shown that cerebral white matter abnormalities are commonly observed in MDC1A patients (Gawlik and Durbeej, 2011). While underlying mechanisms responsible for white matter abnormalities in MDC1A patients remain elusive, it is speculated that deficiency of merosin disrupts the blood–brain barrier and increases water content, thereby leading to abnormal white matter signal intensity (Menezes et al., 2014). In our case, we consistently observed abnormalities in the white matter of parietal and occipital lobes, whereas the cerebellum and corpus arenaceum were normal. The white matter abnormality represents a typical feature of MDC1A compared with other CMD subtypes.

In summary, this case presented typical features of MDC1A, including appendicular hypotonia, high serum CK levels, and white matter abnormalities. Ultimately, she was diagnosed with MDC1A by genetic analysis. Taken together, this case further confirms the heterogeneous clinical spectrum of MDC1A and presents two novel LAMA2 mutations to expand the mutation spectrum of MDC1A.

## Author Contributions

Conceived and designed the experiments: ZX; performed the experiments and wrote the paper: JZ and JT; analyzed the data: DM, JjZ, and JC; contributed reagents/materials/analysis tools: JC, CL, GL, and YW.

## Conflict of Interest Statement

The authors declare that the research was conducted in the absence of any commercial or financial relationships that could be construed as a potential conflict of interest.

## Footnotes

1 http://www.lovd.nl/LAMA2

2 http://cryp-skip.img.cas.cz/

3 http://www.fruitfly.org/about/index.html
